# Cytotoxic and Genotoxic Effects of Pesticide Exposure in Male Coffee Farmworkers of the Jarabacoa Region, Dominican Republic

**DOI:** 10.3390/ijerph15081641

**Published:** 2018-08-03

**Authors:** Hans-Peter Hutter, Abdul Wali Khan, Kathrin Lemmerer, Peter Wallner, Michael Kundi, Hanns Moshammer

**Affiliations:** Department of Environmental Health, Center for Public Health, Medical University Vienna, Kinderspitalgasse 15, 1090 Vienna, Austria; khanzadaamc@yahoo.com (A.W.K.); kathrin.lemmerer@meduniwien.ac.at (K.L.); peter.wallner4@gmail.com (P.W.); michael.kundi@meduniwien.ac.at (M.K.); hanns.moshammer@meduniwien.ac.at (H.M.)

**Keywords:** biomonitoring, coffee plantation, cytotoxic risk, genotoxic risk, occupational health, pesticide, sprayer

## Abstract

Intensive agrochemical use in coffee production in the Global South has been documented. The aim of this study was to investigate cytotoxic and genotoxic effects of pesticide exposure in male farmworkers in the Dominican Republic comparing conventional farming using pesticides to organic farming. Furthermore, feasibility of the buccal micronucleus cytome assay (BMCA) for field studies under difficult local conditions was tested. In a cross-sectional field study, pesticide exposed (sprayers) and non-exposed male workers on coffee plantations were interviewed about exposure history, and pesticide application practices. Buccal cells were sampled, and BMCA was applied to assess potential effects on cell integrity. In total, 38 pesticide-exposed and 33 non-exposed workers participated. Eighty-four and 87%, respectively, of the pesticide-exposed respondents did not use masks or gloves at all. All biomarkers from the BMCA were significantly more frequent among exposed workers—odds ratio for micronucleated cells: 3.1 (95% confidence interval: 1.3–7.4) or karyolysis: 1.3 (1.1–1.5). Buccal cells as sensitive markers of toxic oral or respiratory exposures proved feasible for challenging field studies. Our findings indicate that the impact of pesticide use is not restricted to acute effects on health and wellbeing, but also points to long-term health risks. Therefore, occupational safety measures including training and protective clothing are needed, as well as encouragement towards minimal application of pesticides and more widespread use of organic farming.

## 1. Introduction

Agriculture is the largest employment sector in the Dominican Republic, accounting for 45% of total labour force of the country [1,2]. Like other northern Latin American and Caribbean countries, a major farming sector is coffee cultivation [3]. The majority of these workers in the Dominican Republic (D.R.) are uneducated and poor, and the lack of awareness about pesticide hazards, as well as lack of effective regulations, makes them vulnerable to all types of health hazards from pesticide exposure [4]. Previous studies mostly focused on ecological aspects of farming, and only few studies reported on health hazards in the D.R.—e.g., neurobehavioral disorders [5]. However, little is known about health effects on farmers in the D.R. due to pesticide exposure. Due to large numbers of farmers in the agriculture sector using different pesticides without any preventive measures, it is critical to assess health problems, including early signs of cellular damage.

After the mid-1940s, large numbers of synthetic pesticides were available on the market worldwide [6]. Currently, there are more than 900 different pesticides in over 2600 products on the market [7]. Different morbidities and mortalities are reported to occur as a result of pesticide poisoning, particularly in developing countries [8,9]. Concomitant to acute poisoning, chronic exposure to pesticides can result in a variety of health disorders, e.g., reproductive problems [10,11], and neurodegenerative disorders such as Parkinson’s [12,13], and Alzheimer’s disease [14,15]. Moreover, chronic occupational exposure is reported to cause different site-specific cancers in humans, e.g., brain cancer, blood cancers (leukaemia), Ewing’s sarcomas, kidney cancer, testicular cancer, colon cancer, rectal cancer, endocrine glands, and soft tissue cancers [7,16]. Therefore, many pesticides with known toxic properties have been banned, and their use has been restricted on the market. However, persistent accumulation of some pesticides still poses health risks in numerous ecosystems globally [7]. In countries with poor regulation or control, problematic pesticides banned in the EU or in the United States might still be in use [17,18].

In the D.R., organophosphates (e.g., glyphosate), phenoxy herbicides (e.g., 2,4-dichlorophenoxyacetic acid (2,4-D)), as well as insecticides like pyrethroids, are used extensively [19,20]. Some studies in adults and children have linked organophosphate exposure to lymphoma and leukemia. Furthermore, long-term exposure to low levels of organophosphate pesticides may produce neuropsychiatric symptoms [21]. Based on longer-term toxicity studies, plausible outcomes of pyrethroid exposure in humans would be primarily neurological symptoms [22], which were not addressed in almost all occupational health studies. Furthermore, it has to be noted that the herbicides 2,4-D and glyphosate were classified as possibly carcinogenic to humans (Group 2B) by the IARC [23,24].

In the past, lymphocytes were predominantly used to assess genotoxic effects, reporting an increase in the number of nuclear anomalies predictive of DNA damage [25,26,27,28]. Later on, this approach was extended to exfoliated cells of subjects exposed to pesticides orally or by inhalation, using the buccal micronucleus cytome assay (BMCA) [29,30,31]. For genotoxic nuclear anomalies, the typical biomarker is the increase in frequency of micronuclei (MNi), which are formed as a result of chromosomal breakage or spindle interference during mitosis [32,33]. MNi in exfoliated buccal cells are, like those in lymphocytes, useful biomarkers for determining cancer risks, due to exposure to carcinogens [34,35]. Some studies reported a statistically significant increase in nuclear anomalies, reflecting genotoxicity in workers not using personal protective devices during spraying [36,37,38,39].

Assuming that patterns of use of preventive measures (personal protective devices e.g., masks) for the safer use of pesticides is less than perfect, farmworkers are directly exposing, via inhalation, their buccal mucosa to pesticides during spraying. Furthermore, oral exposure via hand to mouth contact or contaminated nutrition during working hours can occur. Thus, in the study, the frequencies of both cytotoxic and genotoxic nuclear anomalies were determined in exfoliated buccal cells using BMCA. Suitability under field conditions (e.g., non-invasiveness of the procedure, easy slide preparation, simple storage, and easy transport at room temperature), further substantiated BMCA as the preferred technique [33,35]. The aim of the study was to determine both cytotoxic and genotoxic nuclear anomalies associated with exposure to pesticides in exposed workers compared to controls. Furthermore, we assessed the feasibility of the BMCA for field studies under difficult local conditions.

## 2. Material and Methods

### 2.1. Study Area and Subjects

**Figure 1:**
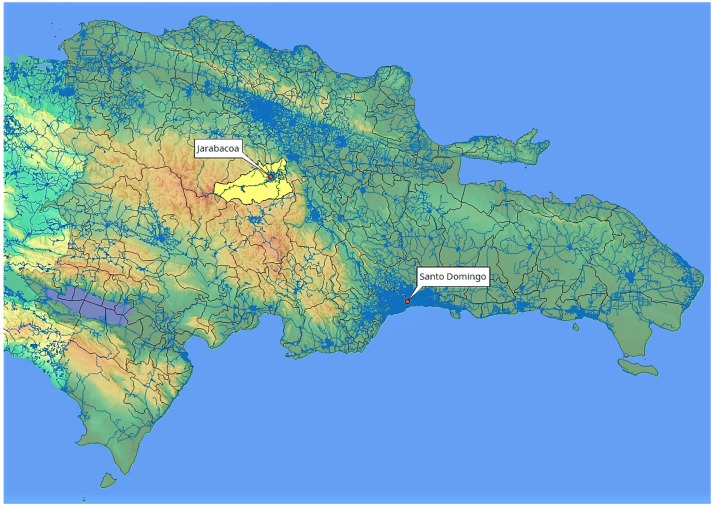
Overview of geographical location of the study site (Jarabacoa, province La Vega).

The complex tropical landscape of the Jarabacoa coffee region in the province La Vega of the D.R. was chosen for our study. Jarabacoa is located in the mountains of the Central Mountain Range between 800 and 1500 m above sea level (Figure 1). This area is well known for coffee plantation (both organic and conventional cultivation) as a significant percentage of coffee production in the region is located there. The region is dominated by agriculture (i.e., coffee, chayote, strawberries) and can be classified as subtropical moist forest, with modest rainfall (1000–1500 mm/year), rich vegetation, and acidic soils [40,41,42].

In total, 38 pesticide-exposed and 33 non-exposed male workers were selected by random choice of the villages, and contacting every farm worker there for participation. Informed consent was obtained from each individual before the research began. The sample size was considered sufficient to detect cytological effects [35]. Exposed participants had to work as sprayers using pesticides for more than 5 years. Furthermore, the selected exposed workers had been using pesticides manually for at least 3 weeks before investigation. The control group also consisted of farmer workers working in the same region but practicing organic farming for at least five years (= non-exposed).

Data regarding demographic variables and indicators of exposure (working conditions etc.) were obtained using a questionnaire adapted to local conditions. Relevant information about the type of pesticide(s) used, duration and intervals of spraying, and personal protective measures, were used by interviewers from the study areas, specifically trained for the project by the research team.

### 2.2. Buccal Cells Micronucleus Assay (BMCA)

In this study, several endpoints were blindly evaluated, which reflected (i) genotoxic effects including micronuclei (MN), total MN (MNi), nuclear buds and broken eggs (BUD), and binucleated cells (BN) as indicators of cytokinetic defects, as well as (ii) cytotoxic effects, including cells with condensed chromatin (CC), karyorrhectic cells (KR), karyolytic cells (KL), and pyknosis (PY). In addition, basal cells were counted to assess proliferative activity.

#### 2.2.1. Buccal Cell Sampling

Buccal cells were collected, and, subsequently, slides were prepared according to the procedure described by Tolbert et al. [29]. The participants were asked to rinse their mouths twice with tap water immediately before the procedure. This was necessary to get rid of food debris in the mouth. The cells were then collected from buccal mucosa of both cheeks using separate moist wooden spatulas for each side. Thereafter, the cell samples were smeared gently on the end of each slide with 2–3 drops of distilled water using a sterile pipette. From each subject, two slides (i.e., one each from the left and the right cheeks) were prepared. The slides were air dried for 10 min, then stored in a dry and dark place, and later on, shipped to Vienna for fixation, staining, and evaluation.

#### 2.2.2. Slide Fixation and Staining

The slides were placed back to back in Coplin jars and fixed with freshly prepared cold methanol and a glacial acetic acid mix (3:1). The slides were air dried at room temperature for 10 min, then stained with 5 M 37% (*v*/*v*) in hydrochloric acid at room temperature for 30 min, according to the established Feulgen technique [30]. The slides were rinsed with running tap water for 3 mins, then stained with Schiff’s reagent (Sigma-Aldrich, Steinheim, Germany) at room temperature and under dark conditions for an additional 90 min. The slides were washed for 5 min with running tap water, counterstained with 0.2% (*w*/*v*) Light Green (Sigma-Aldrich, Steinheim, Germany) cytoplasmic stain for 20 s, and were then washed again with running tap water.

#### 2.2.3. Evaluation of Nuclear Anomalies

The slides were evaluated and scored for nuclear anomalies according to the current standard protocol by Thomas et al. [31]. Before evaluation, the slides were coded by a person not directly involved in evaluation. From each person, ≥2000 buccal cells were evaluated and scored under 400-fold magnification under both bright-field and fluorescence (using a far-red filter) microscope (Nikon Labophot-2, Tokyo, Japan). In the first step, frequencies of basal cells, differentiated cells, and cells with nuclear anomalies were scored in 1000 buccal cells. In the second step, the counting of differentiated cells continued for genotoxic nuclear anomalies (MNi, total MNi, BUD) until the total count of differentiated cells reached 2000. Photographic images of important nuclear anomalies were taken with a digital microscopic camera (Nikon DS-Fii1, Tokyo, Japan). Concomitantly, all anomalies were cross-checked by another experienced scorer.

The range of values for the background counts of these biomarkers show high variability although the ratio of counts for exposed to controls is more stable [43].

### 2.3. Statistical Analyses

As the primary exposure variable, current handling of pesticides was investigated. As potential confounders, age (in years), body mass index (BMI), smoking and tobacco chewing, alcohol consumption, dental X-rays during the last month, and frequency of eating spicy food (in days per week) were considered.

As it is well known that, in particular, damage of buccal cells can arise from ionizing radiation exposure, it is necessary to exclude such conditions. Spicy food might act as such a confounder too. However, the evidence regarding the influence of spicy food is mixed.

Frequency of nuclear anomalies was analysed applying Poisson regression with number of counted cells as offset variable. Overdispersion was assessed for all analyses; however, no significant excess variance was detected. All calculations were done with STATA 13.1 SE (StataCorp, College Station, TX, USA).

*p* values < 0.05 were considered to be statistically significant.

## 3. Results

In total, 38 pesticide-exposed and 33 non-exposed workers participated in the cross-sectional field study. The average ages were 34.6 years (pesticide exposed workers) and 48.5 years (non-exposed workers), respectively. Organic farmers had similar education and dietary habits, as well as smoking, tobacco chewing, and alcohol-drinking habits, compared to pesticide-exposed workers. However, we found significant differences with regard to age, and related to age in number of children and body mass index (Table 1). Therefore, age and BMI were included in the analyses of the BMCA outcomes.

**Figure 2:**
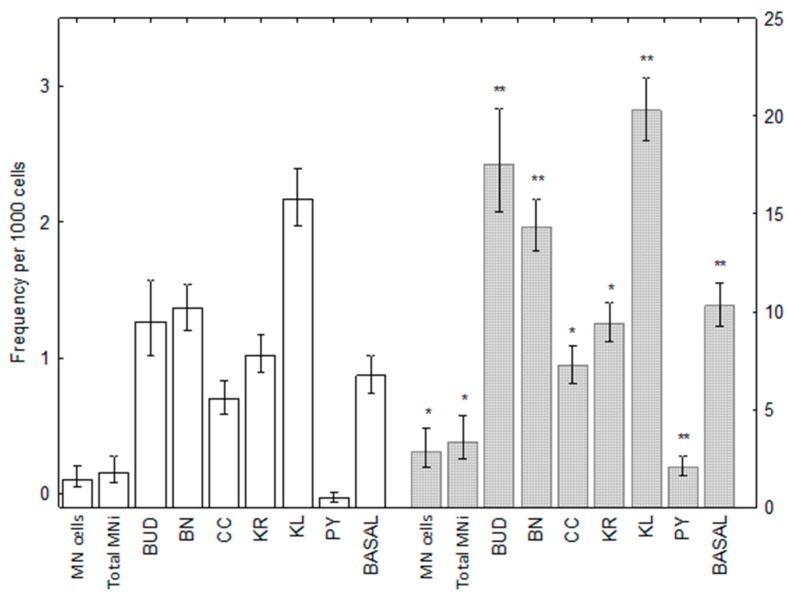
Means and 95% confidence intervals of nuclear anomalies in buccal cells from non-exposed workers (light bars) and pesticide-exposed workers (dark bars). MN cells to BUD: left axis, BN to BASAL: right axis. * *p* < 0.05, ** *p* < 0.01; MN cells: number of micronucleated cells; Total MNi: total number of micronuclei; BUD: nuclear buds & broken eggs; BN: binucleated cells; CC: condensed chromatin; KR: karyorrhexis; KL: karyolysis; PY: pyknosis; BASAL: basal cells.

Figure 2 shows the results of our analysis for nuclear anomalies in pesticide workers relative to controls (corrected for age, tobacco chewing, smoking, BMI, and alcohol consumption): pesticide workers had significantly more cellular anomalies for all endpoints studied. We found odd ratios ranging from 1.2 (karyorrhexis) up to 4.6 (pyknosis) (Table 2).

Pesticide-exposed workers were not only spraying pesticides, but prepared and mixed the pesticides themselves, and were also responsible for disposal. Only one participant was not able to provide information on the type of agrochemicals used. The pesticides mentioned by the other 37 participants were mostly herbicides and fungicides, followed by insecticides. The herbicides applied were in almost all cases organophosphates, predominantly glyphosate. In several cases, the use of paraquat was reported; some participants mentioned the use of 2,4-D (2,4-dichlorophenoxyacetic acid). Among the insecticides, cypermethrin and carbamate were mentioned most often.

During active application (spraying), the majority of the sample reported never used masks or gloves. Only 4–5% of the respondents (pesticide workers) reported using masks/gloves all the time.

One aim of our study was to develop an epidemiological approach which is scientifically sound, non-invasive, feasible under poor conditions (lack of infrastructure), and inexpensive—but techniques must be reliable. We are aware that other different human biomonitoring methods (blood or urine) are providing more detailed information on exposure. However, the efforts (lab equipment and material) and the costs (analysis) would be much higher, and for us, not feasible, especially due to cooling requirements. As an aside, it must be pointed out that the study area was lacking infrastructure (no electricity, no easy access).

Furthermore, it has to be noted that the micronucleus assay has been widely used as an in vivo assay as the most reliable assay to assess the induction of chromosomal aberrations (one of two major endpoints of mutagenicity).

## 4. Discussion

Within the scope of our study, we assessed feasibility of the BMCA for field studies under difficult local conditions and investigated whether long-term application of pesticides are associated with effects on the oral mucosa. The study was conducted in areas with poor infrastructure (almost no electricity) and that were difficult to access, especially in the rainy season. Under these conditions, the micronucleus assay in exfoliated buccal cells was found to be an optimal method to assess potential genotoxic and cytotoxic effects. Exfoliated epithelial cells can be easily collected, even under challenging field conditions. As cytotoxic and genotoxic biomonitoring in humans has been shown to be a useful and feasible tool to estimate the chronic health risks from an exposure to complex mixtures of chemicals, we integrated this non-invasive method into our field study. Although the micronucleus assay is a well-established, standardized test for the detection of chromosomal aberrations, the costs are comparatively low.

We used the BMCA to assess whether the exposure to mixtures of pesticides leads to an increase in cellular anomalies in a group of workers in coffee plantations in the D.R. The selected region for the study is well known for its coffee farming, conventional as well as organic. Additionally, low awareness about pesticide hazards by the workers was assumed, which is common in many producers’ countries [21]. Farmworkers, directly involved in the handling of pesticides, are at a high risk of exposure to pesticides especially due the absence of protective equipment or failure to use it properly [44,45].

Genotoxic anomalies are a first warning sign indicating carcinogenic potential of the exposure. An increased rate of cell anomalies can thus be used to predict increased risks of cancer since buccal cells are of epithelial type. Our results demonstrate that the group of pesticide users exhibits significantly higher rates of nuclear anomalies compared to non-exposed workers. Micronuclei, as genotoxicity markers, increased about 3-fold, and also, cytotoxic endpoints were significantly elevated with a more than 4-fold increase of pyknotic cells. As it is known that there are different confounding factors, such as age, alcohol consumption, and smoking habits [46], and we considered these factors carefully. 

With regard to MN analysis in buccal cells, only few occupational studies with pesticide users have been carried out. Some of them have shown a lack of an increase in MN (e.g., [47]). However, in other previous studies, an increase in cell abnormalities was observed. Benedetti et al. [48] found significant increases in the frequency of MN in soybean workers. Analysis of buccal cells in a group of Malaysian farmers revealed that the frequency of MN was significantly higher in pesticide-exposed farmers as compared to controls (office workers) [49]. Similarly to our outcome, da Silva et al. [50] reported DNA damage in tobacco workers associated with the exposure to herbicides (organophosphates) and insecticides (carbamates). Dutta et al. [51] observed also a higher proportion of micronuclei and cell death parameters in pesticide-exposed tea garden workers.

The vast majority of participants in our study did not use personal protective devices (e.g., masks) during spraying, directly exposing their skin and also the oropharynx during spraying and indirectly, due to lack of proper hand hygiene during eating and drinking. In a study in Polish workers, who were exposed to complex mixtures of pesticides including organophosphates, but used predominantly more than one kind of protective measure, there were no statistically significant differences in the frequencies of cytogenetic damage between exposed and control individuals [47], which may indicate that consequences of exposure on oral mucosa, and maybe the overall respiratory system, can be prohibited by proper protective measures.

The findings of various epidemiological studies on occupational pesticide exposure are not unequivocal. However, these studies still support the conclusion that farm workers exposed to pesticides might have a significantly higher risk of developing cancer like non-Hodgkin lymphomas and leukaemia (e.g., [52,53,54,55]). The International Agency for Research on Cancer (IARC) has classified certain pesticides as human carcinogens, e.g., the herbicide glyphosate and its formulations [24,56], which were used by the majority of participants in our study.

Although we applied rigorous methods, the study has some limitations. We were not able to match non-exposed controls by education and age. Organic farmers were generally older and better educated. Despite control for these variables in the analysis, residual confounding is possible. Furthermore, buccal cells were collected in the field with limited access to cooling. However, cells from both groups of workers were treated equally, and no bias is expected to occur from the difficult field conditions.

## 5. Conclusions

Occupational health standards are poor or poorly controlled in developing countries. Concerning pesticide use, unsafe application techniques, low levels of education and poor or adverse health conditions among workers, and use of pesticides with higher toxic potential, are found [57,58].

Many imported food products consumed in high-income countries are produced in countries in the Global South. Occupational exposure to pesticides in such countries has received almost no attention, while quite high concern is associated with the consumption of potentially contaminated products in high-income countries. There is little awareness in Western high-income countries about the conditions in which the imported food items from the Global South are produced. 

Our results of the micronucleus cytome assays demonstrate impressively that the exposure to a mixture of agrochemicals may lead to long-term health consequences, and suggest that pesticide users might have a higher risk of developing cancer. From an occupational health standpoint, the urgent need for protective measures and enforcement of occupational standards for the affected farm workers must be addressed. Besides implementing more protection measures for the workers, the sale and the use of highly hazardous pesticides should be restricted [59,60]. Moreover, the question arises in which direction agriculture will develop in the future (more pesticide use vs more organic farming). Our significant results demonstrate that further holding onto conventional agriculture under present conditions may be associated with serious adverse health effects of the exposed workers. Therefore, a more restrictive pesticide policy, stronger regulations regarding workers’ protection, and more support for organic farming (by regulatory and structural measures) is of importance.

## Figures and Tables

**Table 1:** Attributes of farmer worker (non-exposed and pesticide exposed workers), mean ± SD or number (percent).

Endpoints	Non-Exposed Workers	Exposed Worker	*p*-Value
Age (years)	48.5 ± 20.7	34.6 ± 14.1	0.004
Body mass index (BMI) (kg/m²)	24.9 ± 3.1	23.4 ± 2.8	0.046
Education			0.228
None	5 (15%)	3 (8%)	
Compulsory	17 (52%)	27 (71%)	
Secondary	11 (33%)	8 (21%)	
Number of children			0.013
0	7 (21%)	21 (55%)	
1	5 (15%)	3 (8%)	
2	8 (24%)	2 (5%)	
3+	13 (39%)	12 (32%)	
Dental X-ray	0 (0%)	3 (8%)	0.243
Tobacco chewing	4 (12%)	9 (24%)	0.237
Smoking	3 (9%)	2 (5%)	0.658
Spicy food (days/week)	0.7 ± 1.5	0.8 ± 1.5	0.563
Pesticide spraying (years)	1.1 ± 5.1	18.3 ± 11.7	<0.001
Spraying (days/week)		1.5 ± 1.5	
Last spraying (days ago)		10.2 ± 6.1	
Wearing gloves (always + half of the time)		4 (11%)	
Wearing masks (always + half of the time)		5 (13%)	

**Table 2:** Odds ratio and 95% confidence intervals for nuclear anomalies in pesticide workers relative to controls corrected for age, BMI, tobacco chewing, smoking, and alcohol consumption.

Endpoints	OR	95% CI	*p*-Value
MN cells	3.098	1.297–7.404	0.011
Total MNi	2.524	1.219–5.226	0.013
Nuclear buds & broken eggs (BUD)	1.916	1.448–2.536	<0.001
Binucleated cells (BN)	1.412	1.207–1.650	<0.001
Condensed chromatin (CC)	1.306	1.054–1.618	0.015
Karyorrhexis (KR)	1.212	1.030–1.426	0.021
Karyolysis (KL)	1.286	1.132–1.462	<0.001
Pyknosis (PY)	4.536	2.517–8.173	<0.001
Basal cells	1.526	1.263–1.844	<0.001
